# The Relationship between Myoglobin, Aerobic Capacity, Nitric Oxide Synthase Activity and Mitochondrial Function in Fish Hearts

**DOI:** 10.3390/antiox10071072

**Published:** 2021-07-03

**Authors:** Lucie Gerber, Kathy A. Clow, William R. Driedzic, Anthony K. Gamperl

**Affiliations:** Department of Ocean Sciences, Memorial University, St. John’s, NL A1C 5S7, Canada; kclow@mun.ca (K.A.C.); driedzic@mun.ca (W.R.D.); kgamperl@mun.ca (A.K.G.)

**Keywords:** mitochondrial respiration, reactive oxygen species, nitric oxide, citrate synthase activity, salmon, sculpin, lumpfish

## Abstract

The dynamic interactions between nitric oxide (NO) and myoglobin (Mb) in the cardiovascular system have received considerable attention. The loss of Mb, the principal O_2_ carrier and a NO scavenger/producer, in the heart of some red-blooded fishes provides a unique opportunity for assessing this globin’s role in NO homeostasis and mitochondrial function. We measured Mb content, activities of enzymes of NO and aerobic metabolism [NO Synthase (NOS) and citrate synthase, respectively] and mitochondrial parameters [Complex-I and -I+II respiration, coupling efficiency, reactive oxygen species production/release rates and mitochondrial sensitivity to inhibition by NO (i.e., NO IC_50_)] in the heart of three species of red-blooded fish. The expression of Mb correlated positively with NOS activity and NO IC_50_, with low NOS activity and a reduced NO IC_50_ in the Mb-lacking lumpfish (*Cyclopterus lumpus*) as compared to the Mb-expressing Atlantic salmon *(Salmo salar)* and short-horned sculpin *(Myoxocephalus scorpius*). Collectively, our data show that NO levels are fine-tuned so that NO homeostasis and mitochondrial function are preserved; indicate that compensatory mechanisms are in place to tightly regulate [NO] and mitochondrial function in a species without Mb; and strongly suggest that the NO IC_50_ for oxidative phosphorylation is closely related to a fish’s hypoxia tolerance.

## 1. Introduction

Nitric oxide (NO) is a gasotransmitter that is normally produced in a reaction catalyzed by the enzyme NO synthase (NOS) which requires O_2_ to oxidize L-arginine, and several cofactors such as tetrahydrobiopterin (H_4_B) and nicotinamide adenine dinucleotide phosphate (NADPH) [1]. However, tissue NO levels are also determined by reactions involving metal-containing oxygen binding proteins (e.g., myoglobin, Mb). Under normoxic conditions, Mb bound to oxygen (oxyMb) reacts with NO forming metMb and nitrate, whereas under hypoxic/anoxic conditions deoxyMb acts as a nitrite reductase and reduces nitrite to NO [2]. In the heart, at the level of the mitochondrion, this dynamic NO-Mb cycle drives reversible adjustments in respiration, and this optimizes O_2_ utilization based on intracellular O_2_ tension while at the same time controlling the level of free radicals [3,4,5]. NO can reversibly bind to complex-IV (CIV) and result in the S-nitrosation of complex-I (CI), and thus, modulate both mitochondrial O_2_ consumption and the production of reactive oxygen species (ROS) [6,7]. For example, S-nitrosation of CI was recently shown to decrease both ROS production and CI activity in cardiac mitochondria of the freshwater turtle *Trachemys scripta* [8]. Hence, the absence of the NO scavenger Mb may have important implications for ROS production and CI capacity. The dynamic interaction between NO and Mb signaling with regard to cardiovascular homeostasis has received considerable attention [4,9,10,11]. Mb is highly expressed in the hearts of many fishes [12] and appears to be the predominant nitrite reductase in the heart of some fish [13]. Yet, the complex relationship between these two chemical entities in controlling O_2_ and NO homeostasis (1) is not fully understood, notably at the level of the mitochondrion; and (2) is challenged by the existence of species that naturally lack the O_2_ carriers hemoglobin (Hb) and/or Mb [14,15].

The lack of Mb, and therefore the loss of the principal NO degradation mechanism, in some fish hearts [13] may have critical implications for mitochondrial and cardiovascular regulation [16]. Indeed, mitochondrial respiration in the heart of some fish species has been shown to be under a tone from NO [14]. Species that have lost the Hb- and/or Mb- genes as part of their evolutionary history offer a unique opportunity to study adaptations to the absence of these major O_2_ carriers and NO regulators [17,18]. The cardiovascular system of globin- (Mb and/or Hb) lacking/poor fishes has received considerable attention, and comparative studies with globin-expressing/rich fishes have revealed unique cardiovascular changes that compensate for the lack of O_2_ oxygen carriers and/or a lower blood oxygen-carrying capacity [17,19,20,21,22,23]. It has been suggested that some of these modifications in the cardiovascular system were triggered by the lack of these principal NO scavengers [4,24]. Indeed, the loss of circulating Hb was associated with the activity and abundance of NOS in various tissues, and higher basal NO metabolites (NOx, nitrite and nitrate) in the plasma of Antarctic Hb^−^ icefishes as compared to Hb^+^ species [23,25]. Nonetheless, research is needed on non-Antarctic species, notably on hemoglobin-rich teleosts lacking Mb (i.e., Hb^+^ Mb^−^ fishes) [18,26,27] before we can understand the complex role that Mb plays in both NO homeostasis, and in regulating mitochondrial function, in the heart [5].

Recently, we have been able to measure the [NO] that reduces mitochondrial respiration by 50% (i.e., mitochondrial sensitivity to NO; its NO IC_50_), and this parameter appears to display considerable plasticity when fishes respond to changes in energy demand or O_2_ tension. For example, sablefish (*Anoplopoma fimbria*) cardiac mitochondria were more sensitive to inhibition by NO after acclimation to low O_2_ conditions (i.e., the NO IC_50_ decreased [28]), whereas in Atlantic salmon (*Salmo salar*), cardiac mitochondria were less sensitive to inhibition by NO after acclimation to elevated temperature (20 °C; i.e., the NO IC_50_ increased [29]). This plasticity in mitochondrial sensitivity to NO may play an important role in preserving both NO and O_2_ homeostasis, and in protecting cardiac aerobic metabolism in fishes.

Based on the above information/data, we hypothezised: (1) that the loss of Mb has led to adjustments of the NOS/NO system (i.e., NOS activity and mitochondrial NO IC_50_) to optimize the utilization of O_2_ and mitochondrial function in cardiac tissues; and (2) that a positive correlation exists between mitochondrial NO IC_50_ and Mb levels in the fish heart. Collectively, these changes would allow Mb-less fish to overcome the lack of Mb by slowly generating NO (i.e., reducing NOS activity) and quickly inhibiting mitochondrial respiration (i.e., by increasing the sensitivity to inhibition by NO). These two compensatory mechanisms may allow fish without (or with lower) cardiac Mb levels to rely on NOS-generated NO to maintain O_2_ and NO gradients at the level of the mitochondria, and to avoid high levels of basal NOx in the absence of Mb.

In this study, we used the hearts of three red-blooded (Hb^+^) fishes [one Mb-lacking (Mb^−^) species (lumpfish; *Cyclopterus lumpus*) and two Mb-expressing (Mb^+^) fish species, the Atlantic salmon (*Salmo salar*) and short-horned sculpin (*Myoxocephalus scorpius*)], which have different ecophysiological traits (including activity levels) to: (1) determine cardiac mitochondrial O_2_ consumption, ROS production and mitochondrial sensitivity to inhibition by NO (NO IC_50_); (2) measure the activity of NOS and citrate synthase as indicators of NO production and cellular oxidative capacity, respectively; and (3) examine the relationship(s) between cardiac Mb level, NOS activity and NO IC_50_ with regard to the regulation of cardiac mitochondrial function. This comparative analysis provides valuable insights into how Mb tissue level, NO production and mitochondrial sensitivity to NO interact to adjust mitochondrial function and cardiac metabolism to ensure sustained function, and to avoid the damaging effects of excess ROS production.

## 2. Materials and Methods

### 2.1. Experimental Animals

Atlantic salmon (*Salmo salar*, Hb^+^ Mb^+^; from Cape d’Or Sustainable Seafood Inc., NS, Canada) and lumpfish (*Cyclopterus lumpus*, Hb^+^ Mb^−^; reared at the Dr. Joe Brown Aquatic Research Building, JBARB) were held in the JBARB at the Ocean Sciences Centre (OSC, Memorial University of Newfoundland). Short-horned sculpin (*Myoxocephalus scorpius*, Hb^+^ Mb^+^) were collected locally and held in the OSC’s aquatic facilities for 3 weeks before experiments. Fish were kept in aerated seawater at 7 ± 1 °C and provided with a 12 h light: 12 h dark photoperiod. Atlantic salmon and lumpfish were hand fed a commercial marine fish diet (Europa; Skretting Inc., St Andrews, NB, Canada) at ~1–1.5% body mass day^−1^, whereas short-horned sculpins were hand fed chopped herring to satiation bi-weekly.

### 2.2. Mitochondrial Isolation

Isolation of mitochondria was performed as described previously [28]. In brief, after the fish was killed with a blow to the head and the body mass was taken, the heart was dissected out and flushed with 10 mL of PBS (in mmol L^−1^: 137 NaCl, 2.7 KCl, 1.8 KH_2_PO_4_, and 10 Na_2_HPO_4_; pH 7.4 at 20 °C) to remove the blood. The ventricle was then cut in half, rinsed with PBS, blotted dry and weighed. Half of the ventricle was snap-frozen in liquid nitrogen and kept at −80 °C for later determination of enzymatic activities and myoglobin content. The other half was homogenized in ice-cold isolation medium (in mmol L^−1^: 230 mannitol; 75 sucrose; 20 HEPES; 1 EGTA; pH 7.4 at 20 °C) using six passes of a loose-fitting motor driven Teflon pestle. The ventricle homogenate was centrifuged at 800× *g* for 10 min at 4 °C and the resulting supernatant was centrifuged at 8000× *g* for 10 min at 4 °C to pellet the mitochondria. The pellet was then washed twice by gentle re-suspension in ice-cold isolation medium containing 10 mg mL^−1^ of BSA (CAS 9048–46-8) and centrifuged at 8000× *g* for 10 min at 4 °C. The final pellet was re-suspended in ice-cold respiration medium (in mmol L^−1^: 160 KCl; 30 HEPES; 10 KH_2_PO_4_; 1.5 MgCl_2_; 1 EDTA (2 EDTA for sculpin); 10 mg mL^−1^ BSA (fatty acid free), pH 7.4 at 20 °C) and the mitochondria were kept on ice for 30 min to rest before use. The protein content of the mitochondrial suspensions was determined using the Bradford assay (Thermo Fisher Scientific, Waltham, MA, USA).

### 2.3. Mitochondrial Physiology

The experimental protocols detailed below are shown in Supplementary Figures S1 and S2. The mitochondrial parameters were measured via high-resolution respirometry using Oroboros Oxygraph-2k instruments (O_2_k-system, Innsbruck, Austria) and following standard operating procedures (O_2_k-SOPs; [30,31]).

**Experiment** **1.** *Measurement of coupled mitochondrial respiration and ROS production/release rate.*

In *Experiment 1* (see Supplementary Figure S1), we measured the ROS production/release rate along with mitochondrial respiration. The ROS production/release rate was estimated by measuring extra-mitochondrial H_2_O_2_ using the constituents of the Amplex^®^ UltraRed [AmR, Thermo Fisher Scientific, Waltham, MA, USA) detection system (AmR 10 µmol L^−1^, horseradish peroxidase (HRP) 3 U mL^−1^, and superoxide dismutase (SOD) 25 U mL^−1^]. The ROS signal was calibrated following standard operating procedures (O_2_k-SOPs; [32,33]), and with the signal amplification and LED intensity adjusted to 1000 and 500 mV, respectively. We injected the cardiac mitochondrial suspensions (0.25 mg protein mL^−1^) into 100% air-equilibrated chambers filled with 2 mL of respiration medium at 12 °C. We followed the SUIT protocol described elsewhere [29], with a few modifications. First, we fueled the electron transport system (ETS) with the complex-I (CI) substrates pyruvate (10 mmol L^−1^), glutamate (15 mmol L^−1^) and malate (2 mmol L^−1^) and a suboptimal concentration of ADP (100 μmol L^−1^). Thereafter, we added an optimal concentration of ADP (250 μmol L^−1^) to measure maximal oxidative phosphorylation (OXPHOS, State 3) respiration via CI (OXPHOS-I). After the depletion of ADP, we measured leak respiration (LEAK, State 4) via CI (LEAK-I). We then added the complex-II substrate succinate (5 mmol L^−1^) and an optimal concentration of ADP (250 μmol L^−1^) to measure maximal OXPHOS respiration via CI+II (OXPHOS-I+II). After the depletion of ADP, we measured leak respiration rate via CI+CII (LEAK-I+II). These measurements also allowed for the calculation of mitochondrial coupling (i.e., respiratory control ratio, RCR = State 3/State 4) and efficiency (i.e., estimation of phosphorylation to oxidation, P:O ratio = [ADP_injected_]/[O_consumed_]) with both CI- and CI+II-substrates in the presence of 250 μmol L^−1^ ADP. Before terminating the experiment, an excess amount of ADP (500 μmol L^−1^) was added and used as a control for *Experiment 2* (see below); i.e., to compare maximal OXPHOS respiration in the absence and presence of NO.

**Experiment** **2.** *Measurement of mitochondrial sensitivity to NO.*

In *Experiment 2* (see Supplementary Figure S2), we connected NO sensors (ISO-NOP, World Precision Instruments, Sarosota, FL, USA) to the Oroboros Oxygraph-2k following standard operating procedures (O2k-SOP; [34]) to determine the concentration of NO in the chamber that inhibited 50% of maximal mitochondrial respiration (i.e., the NO IC_50_ to determine mitochondrial sensitivity to NO). We calibrated the NO sensors following the manufacturer’s recommendations [WPI instruction manual, *Calibration of NO Sensor by Decomposition of SNAP–Method 2*; see [28] for details]. We then followed the SUIT protocol described above (in *Experiment 1*) with a few modifications to determine the mitochondria’s sensitivity to NO (i.e., the NO IC_50_). In *Experiment 2*, after adding an excess amount of ADP (1.2 mmol L^−1^), the NO donor PAPANONOate (15 μmol L^−1^; prepared immediately prior use, Cayman Chemical, Ann Arbor, MI, USA) was added at ∼60% air saturation to initiate NO release (at ∼15 nmol min^−1^) and gradually inhibit maximal OXPHOS-I+II respiration. Once maximal OXPHOS-I+II respiration was inhibited by ∼60%, 2-phenyl-4, 4, 5, 5,-tetramethylimidazoline-1-oxyl 3-oxide (PTIO 0.02 mmol L^−1^) was added to scavenge NO and stop the PAPANONOate-mediated NO release. This reversal of NO’s inhibition of respiration allowed maximal OXPHOS-I+II respiration to be re-established, and the integrity of the outer and inner mitochondrial membranes were then assessed with cytochrome c (10 μmol L^−1^) and NADH (0.5 mmol L^−1^). Before analysis, the recordings of NO concentration were corrected for baseline and zero drift.

### 2.4. Nitric Oxide Synthase Activity

Nitric oxide synthase catalyzes the oxidation of L-arginine to form NO and L-citrulline [35]. In this assay, NOS activity was quantified spectrophotometrically by utilizing the reaction of NO with oxyMb to form metMb. The oxidation of the ferroheme to ferriheme was measured as an increase in absorbance at 404 nm as previously described [36]. The slope of this relationship was used to calculate the amount of NO generated per minute, and the extinction coefficient of 28.1 mmol^−1^ cm^−1^ was used to calculate NOS activity in both ventricular homogenates and mitochondrial suspensions. The extinction coefficient was determined by making a standard curve with variable proportions of oxidized/reduced Mb. This assay procedure included taking previously snap-frozen ventricles and homogenizing them in 4 volumes of homogenization buffer [in mmol L^−1^: 50 Tris, 1 EGTA, 1 EDTA, 1:100 Protease Inhibitor Cocktail (P2714)]. The ventricle homogenate and thawed mitochondrial suspensions were then centrifuged at 12,000× *g* for 10 min and the supernatant collected. In some cases, due to the limited amount of thawed mitochondrial suspensions, supernatants from 2 to 3 individuals had to be combined prior to the measurement of NOS activity. NOS assays were performed at room temperature (22 °C) using a 96-well microplate reader (SpectraMax M2e, Molecular Devices, Sunnyvale, CA, USA). The assay buffer consisted of basic medium (in mmol L^−1^: 100 HEPES, 1 MgCl_2_ and 1 CaCl_2_, pH 7.4 at 20 °C) with 100 µmol L^−1^ of NADPH, 10 µmol L^−1^ of DTT, 25 µmol L^−1^ of oxyMb and 10 µmol L^−1^ of BH_4_. Supernatants were added to the assay mixture and the kinetics were read for 15 min before the reaction was initiated with 30 µmol L^−1^ of L-arginine. The difference in the slopes before and after L-arginine was calculated, and NOS activity was determined as described above. NOS activities for the ventricular homogenates and mitochondrial suspensions were expressed as nmol min^−1^ g tissue^−1^ and nmol min^−1^ mg protein^−1^, respectively. The oxyMb in the assay buffer was prepared as described previously [37], with a few modifications. A solution of 1 mmol L^−1^ of Mb (from equine skeletal muscle, M0630, Sigma-Aldrich, Mississauga, ON, Canada) was dissolved in basic assay medium. The Mb solution was then reduced by adding 1 mg mL^−1^ of sodium hydrosulfite. The sodium hydrosulfite was then removed by running 2.5 mL aliquots down a PD-10 desalting column packed with Sephadex G-25 medium (GE Healthcare, Waukesha, WI, USA) equilibrated with the basic assay medium. The final concentration of Mb was confirmed by reading the absorbance at 580 nm, and using the extinction coefficient of 14.6 mmol^−1^ cm^−1^ [38].

### 2.5. Citrate Synthase Activity

Citrate synthase (CS) activity was measured using the method described previously [39]. Briefly, a snap-frozen piece of ventricle was homogenized in ice-cold homogenization buffer (25 mmol L^−1^ HEPES, 2 mmol L^−1^ EDTA and 0.5% Triton X-100, pH 7.0 at 20 °C) using a Polytron homogenizer (Kinematica Inc., Bohemia, NY, USA). The resulting homogenate was centrifuged at 2000× *g* for 5 min at 4 °C, and CS activity was measured at room temperature (22 °C) using a 96-well microplate reader (SpectraMax M2e). Ventricular CS activities were expressed as µmol min^−1^ g tissue^−1^.

### 2.6. Myoglobin Content

Myoglobin content [Mb] in the ventricle was measured as described previously [40], with a few modifications. Briefly, a snap-frozen piece of ventricle was homogenized (in 25 mmol L^−1^ HEPES and 2 mmol L^−1^ EDTA) using a Polytron homogenizer and centrifuged at 10,000× *g* for 15 min. The supernatant was then collected, and 1 mg mL^−1^ of sodium hydrosulfite was added to reduce all the Mb in the sample. A wavelength scan from 450 to 600 nm was conducted, and the absorbance at the peak of 580 nm recorded. [Mb] was calculated as the ΔABS from the peak to an interpolated baseline. [Mb] was expressed as nmol g tissue^−1^ using an extinction coefficient of 14.6 mmol^−1^ cm^−1^.

### 2.7. Chemicals

All chemicals used in the above procedures were purchased from Sigma-Aldrich (Mississauga, ON, Canada), unless stated otherwise in the method section.

### 2.8. Statistical Analyses

Differences between groups were analyzed using one-way ANOVAs (except for the data for the NO inhibition curves where a two-way ANOVA was used) followed by Tukey’s HSD multiple comparison tests. In addition, linear pairwise regressions and non-linear curve fitting were used to examine the relationships between a number of the measured parameters. These analyses were performed using Prism 8 software (GraphPad Inc., La Jolla, CA, USA). In all cases, a *p* < 0.05 was considered significant.

## 3. Results

### 3.1. Ventricular Characteristics

Relative ventricular mass (RVM) was ~2-fold higher in sculpin (~0.15%) compared to salmon and lumpfish (~0.075–0.079%; Table 1). The Mb content of the ventricle in salmon and sculpin was comparable (~26.5 and 20 nmol g tissue^−1^), whereas this parameter was not detectable in the ventricle of lumpfish (Table 1). Finally, ventricular CS activity was significantly different between all three species (Table 1). The highest CS activity was measured in salmon (6.5 ± 0.3 µmol g tissue^−1^ min^−1^), with values for the sculpin and lumpfish of 5.1 ± 0.2 and 3.8 ± 0.2 µmol g tissue^−1^ min^−1^, respectively.

### 3.2. Mitochondrial Respiration and Coupling

**Figure 1:**
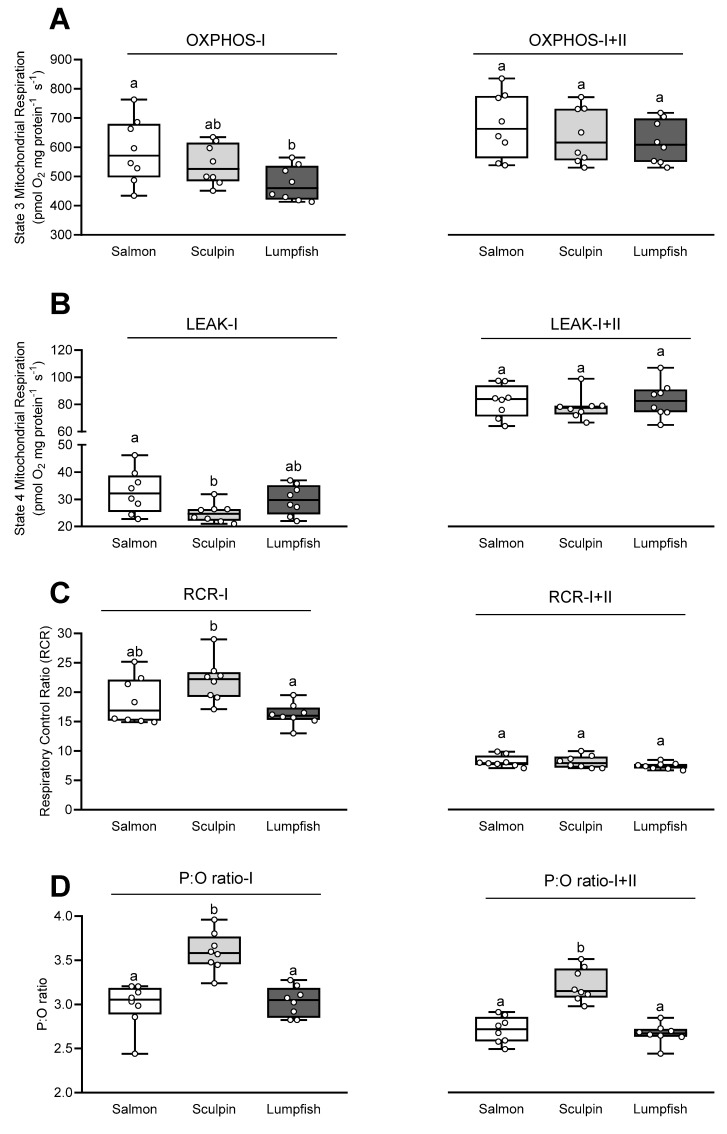
Respiration rates and respiratory control ratios (RCRs) for cardiac mitochondria of Atlantic salmon, short-horned sculpin and lumpfish. (**A**) Oxidative phosphorylation (OXPHOS, State 3) respiration rates and (**B**) LEAK respiration rates (State 4) measured with the complex-I (-I) substrates pyruvate, glutamate and malate, and the complex-I+II (-I+II) substrates pyruvate, glutamate, malate and succinate. (**C**) The resulting RCR (State 3/State 4 values) and (**D**) estimated P:O ratio ([ADP_injected_]/[O_consumed_]). Values without a letter in common are significantly (*p* < 0.05) different; one-way ANOVAs followed by Tukey’s HSD multiple comparison tests. Values are displayed in box-and-whisker-plots; the horizontal line in each box indicates the median value, whereas the top and bottom of the box represent the 25th and 75th percentiles respectively, and the whiskers indicate the highest and lowest values recorded. Symbols represent individual values (*n* = 8 per group).

OXPHOS-I (State 3) respiration was ~20% lower in lumpfish as compared to salmon, whereas the OXPHOS-I respiration for sculpin fell between those for these two species (Figure 1A). While LEAK-I (State 4) respiration was ~25% lower in sculpin as compared to salmon, this parameter in lumpfish was not significantly different from that measured in either of the two other species (Figure 1B). In contrast, the OXPHOS-I+II and LEAK-I+II respiration were comparable between all species (Figure 1A,B). The species-specific differences in OXPHOS-I and LEAK-I resulted in ~25% lower respiratory control ratio values (RCR-I) in lumpfish (~16) as compared to sculpin (~22), whereas the value for salmon was intermediate and not significantly different (~18.5) (Figure 1C). RCR-I+II values were not significantly different between species (~7.5–8; Figure 1C). With regards to P:O ratios for mitochondrial oxidative phosphorylation, values were ~3.0–3.5 with CI substrates and 2.5–3 with CI+II substrates, and ~20% higher in short-horned sculpin as compared to salmon and lumpfish (Figure 1D).

### 3.3. Mitochondrial Reactive Oxygen Species (ROS) Production/Release Rate

**Figure 2:**
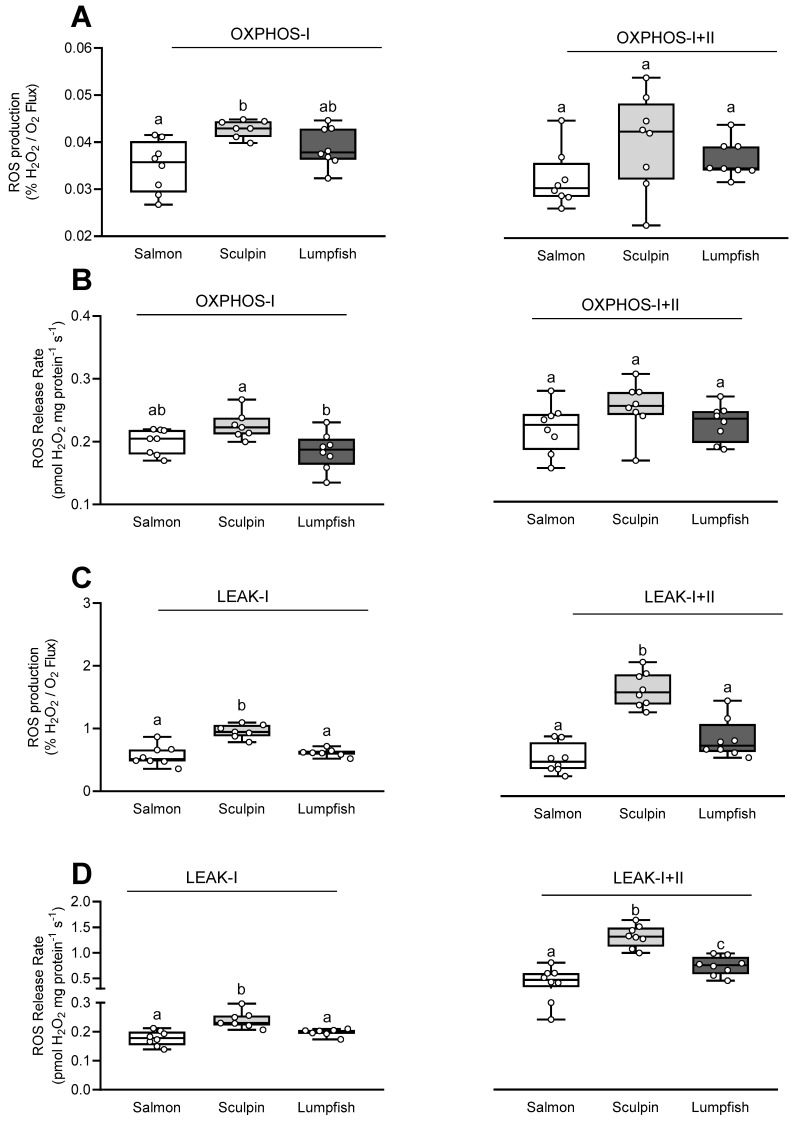
Reactive oxygen species (ROS) production for cardiac mitochondria of Atlantic salmon, short-horned sculpin and lumpfish. The ROS production/release rate during (**A**,**B**) oxidative phosphorylation (OXPHOS) and (**C**,**D**) LEAK respiration with CI and CI+II substrates, and expressed either as a percentage of mitochondrial O_2_ consumption (% H_2_O_2_ flux/O_2_ flux) (**A**,**C**) or as pmol H_2_O_2_ mg protein^−1^ s^−^^1^ (**B**,**D**). Other details are as in Figure 1 (*n* = 7–8 per group). Values without a letter in common are significantly (*p* < 0.05) different.

The ROS production/release rate (estimated as% H_2_O_2_ flux/O_2_ flux) during OXPHOS-I was ~25% higher in sculpin as compared to salmon, whereas the ROS production/release rate for lumpfish fell between those for salmon and sculpin (Figure 2A). In addition, when expressed as pmol H_2_O_2_ mg protein^−1^ s^−1^, sculpin had the highest ROS production/release rate during OXPHOS-I (Figure 2B). This parameter was ~10% higher (but not significantly; *p* = 0.12) as compared to salmon and ~20% greater than that in lumpfish (*p* = 0.01; Figure 2B). In contrast, the ROS production/release rate was comparable in all species during OXPHOS-I+II (Figure 2A,B).

The ROS production/release rate relative to O_2_ flux (respiration rate) during both LEAK-I and -I+II was also significantly higher (by 65% and 90–180%, respectively) in sculpin as compared to both salmon and lumpfish (Figure 2C,D). Further, when expressed relative to respiration (i.e., in pmol H_2_O_2_ mg protein^−1^ s^−1^), the ROS production/release rate during LEAK-I+II was significantly different between all three species (Figure 2D). The highest rate was measured in sculpin and lowest in salmon, while the ROS production/release rate for lumpfish fell in between.

### 3.4. Mitochondrial Integrity and Permeability

**Figure 3:**
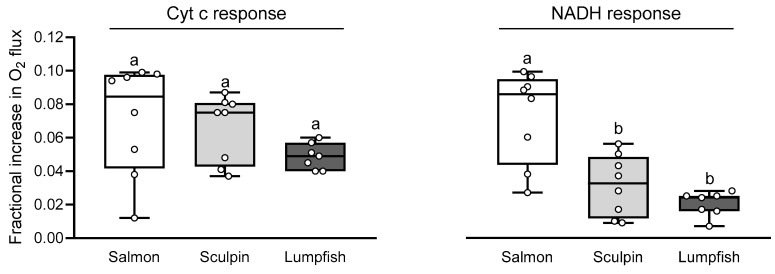
The fractional increase in OXPHOS-I+II respiration of Atlantic salmon, short-horned sculpin and lumpfish cardiac mitochondria following the addition of cytochrome c (Cyt c) and NADH. An increase in O_2_ flux above 0.1 (10%) would suggest a loss of membrane integrity. Other details are as in Figure 1 (*n* = 7–8 per group). Values without a letter in common are significantly (*p* < 0.05) different.

The fractional increase in OXHOS-I+II following the addition of Cyt c and NADH was never higher than 10% (i.e., < 0.1), and this indicates that there was no inner or outer mitochondrial membrane damage/loss of integrity in any of the mitochondrial preparations (Figure 3). The increase in OXPHOS-I+II respiration following Cyt c was comparable in all species (5–7%). However, the increase in OXPHOS-I+II respiration following NADH addition was lower in sculpin and lumpfish (2–3%) as compared to that measured in salmon (7%), and this suggests that salmon had the leakiest inner membrane amongst the three species.

### 3.5. Mitochondrial Sensitivity to NO

**Figure 4:**
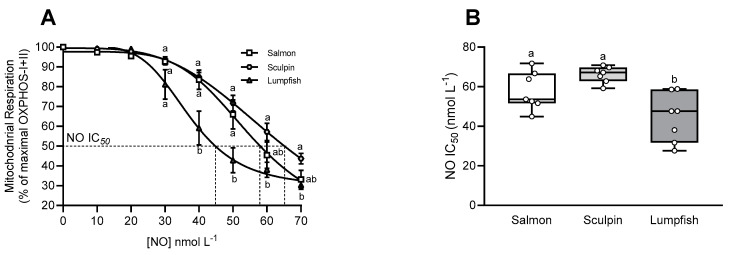
Effect of nitric oxide (NO) on OXPHOS-I+II respiration in cardiac mitochondria of Atlantic salmon, short-horned sculpin and lumpfish. (**A**) Inhibition of mitochondrial respiration by increasing concentration of NO in the chamber (expressed as a percentage of maximal OXPHOS-I+II). Values are means ± s.e.m, and values without a letter in common are significantly different (*p* < 0.05) at a specific [NO]. Two-way ANOVA followed by Tukey’s HSD multiple comparison tests. (**B**) Concentration of NO at which mitochondrial OXPHOS-I+II respiration was half of maximal (NO IC_50_). Other details are as in Figure 1 (*n* = 7 per group).

The release of NO in the chamber gradually inhibited mitochondrial OXPHOS-I+II respiration in all species (Figure 4A). Cardiac mitochondrial OXPHOS-I+II respiration in lumpfish was the most sensitive to inhibition by NO (Figure 4A,B). The NO IC_50_ value (44.3 ± 4.6 nmol L^−1^) for this species was ~35% lower as compared to that measured in salmon (57.9 ± 3.6 nmol L^−1^) and sculpin (66.16 ± 1.5 nmol L^−1^) at the same O_2_ level (100–115 µmol L^−1^ O_2_) (Figure 4B).

### 3.6. Ventricular and Mitochondrial NOS Activity

Ventricular NOS activity in lumpfish (2.2 ± 0.15 nmol g tissue^−1^ min^−1^) was lower (by ~35–45%) as compared to that measured in salmon and sculpin (3.3 ± 0.21 and 3.9 ± 0.27 nmol g tissue^−1^ min, respectively; Table 2). Mitochondrial NOS activity was also lowest in lumpfish (0.04 ± 0.004 nmol mg protein^−1^ min^−1^). However, that for sculpin (0.10 ± 0.02 nmol mg protein^−1^ min^−1^) was intermediate between that measured for lumpfish and salmon (0.15 ± 0.01 nmol mg protein^−1^ min^−1^).

### 3.7. Relationships between Mb Content, Aerobic Metabolism and the NOS/NO System

**Figure 5:**
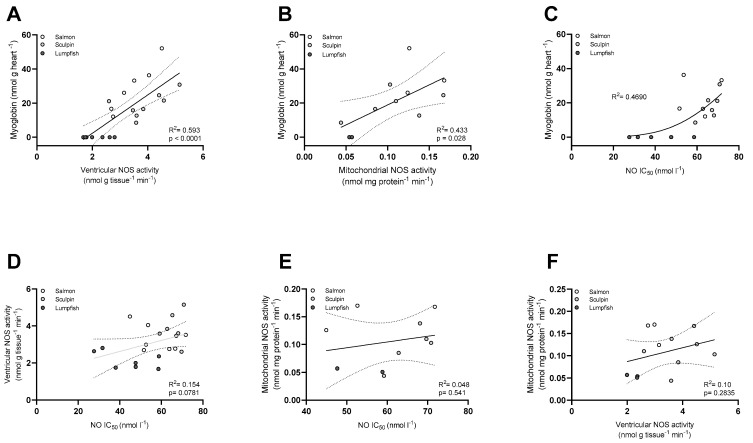
Relationships between heart myoglobin content and the NO/NOS system of Atlantic salmon, short-horned sculpin and lumpfish. Relationship between myoglobin and (**A**) ventricular NOS activity, (**B**) mitochondrial NOS activity and (**C**) mitochondrial sensitivity to inhibition by NO (NO IC_50_). (**D**) Relationship between ventricular NOS activity and NO IC_50_. Relationship between mitochondrial NOS activity and (**E**) NO IC_50_ and (**F**) ventricular NOS. The solid line represents the overall linear pairwise regression and the dotted lines represent the 95% confidence limits of the fitted line (except in Panel C where the solid line represents the best non-linear fit). Circles represent individual values. Values are means ± s.e.m., *n* = 2–7 per group; pooled samples and unpaired values were excluded from these analyses, explaining the lower number of data points for several parameters/groups. Note in Figure 5C: the salmon with the highest Mb content (52 nmol g heart^−1^) was identified as an outlier when fitting the non-linear regression, and was excluded from this analysis. A *p* < 0.05 was considered significant.

Linear pairwise regressions were used to examine the relationships between a number of the measured parameters of the NOS/NO system (NOS activity, NO IC_50_) and Mb content. We found a positive relationship between Mb level in the heart and NOS activity measured in ventricular homogenates and mitochondrial suspensions (R^2^ = 0.59, *p* < 0.0001 and R^2^ = 0.43, *p* = 0.028, respectively; Figure 5A,B). A positive relationship was also found between Mb and mitochondrial NO IC_50_ when fitting the data with a non-linear regression (R^2^ = 0.47; Figure 5C). In contrast, there was only a weak relationship between the mitochondrial NO IC_50_ and NOS activity measured in the ventricle homogenates (R^2^ = 0.15, *p* = 0.0781; Figure 5D). Furthermore, NOS activity measured in the mitochondrial suspensions did not correlate with either the mitochondrial NO IC_50_ or with ventricular NOS activity (R^2^ = 0.048, *p* = 0.54 and R^2^ = 0.10, *p* = 0.28, respectively; Figure 5E,F). Interestingly, we also found a negative relationship between CS activity (an indicator of the aerobic metabolism) and RVM and the mitochondrial P:O ratio (R^2^ = 0.3540, *p* = 0.022 and R^2^ = 0.37, *p* = 0.0017, respectively; Supplementary Figure S3).

## 4. Discussion

Red-blooded fishes without (or differing in) Mb expression are interesting comparative models for investigating the unique physiological traits that have evolved in species that lack the primary degradative pathway for NO in the heart, but retain this pathway in the circulatory system (i.e., Hb). In this study, we compared mitochondrial function and oxidative capacity in the hearts of three species of red-blooded fish that differ in Mb content (Hb^+^ Mb^+^ vs. Hb^+^ Mb^−^) and in physiological traits (e.g., low activity vs. high activity) to examine the role played by Mb in NO and O_2_ homeostasis within cardiac mitochondria and the myocardium.

### 4.1. Myoglobin Content

The [Mb] in the heart of teleost fishes varies over a considerable range (0 to 488 nmol g^−1^; [12]). The Mb content in the ventricle of Atlantic salmon and short-horned sculpin (26.5 and 20 nmol g tissue^−1^, respectively; Table 1) fell toward the low end of this range, and was quite variable. For example, [Mb] in our salmon ranged from 12 to 52 nmol g tissue^−1^. Variability in heart [Mb] has previously been reported in Atlantic salmon with regards to the region of the heart assayed (spongy vs. cortical), and fish size and maturity (larger vs. smaller hearts, and adult vs. immature salmon) [41]. Specifically, these authors reported 82% higher [Mb] in the spongy myocardium than in the cortical myocardium [42], and higher [Mb] in larger hearts from bigger salmon. In addition, [Mb] was 66% higher in the spongy myocardium of adults than in immature salmon (whose hearts consist mostly of spongy myocardium; [43]). The intraspecific variation in [Mb] observed here may be explained by the uneven distribution of Mb in the heart of adult salmon and that we only used a small piece of ventricle for the determination of [Mb]. No values of [Mb] in short-horned sculpin were found in the literature. However, the values reported here were lower than those reported for another sculpin species (*Myoxocephalus octodeeimspinosu*; 76 nmol g^−1^, [21]. The variation in [Mb] among sculpin species is in line with variation in traits related to O_2_ extraction capacity reported among eleven sculpin and related to a species’ hypoxia tolerance [44]. Most importantly, myocardial [Mb] were substantially greater in the Atlantic salmon and short-horned sculpin as compared to the lumpfish where it was not detectable; and this is consistent with previous studies [20,21]. The absence vs. presence of Mb allowed us to study the putative relationships between loss of Mb and the NOS/NO system in the ventricle of red-blooded fishes, and its consequences for mitochondrial function.

### 4.2. Relative Ventricular Mass

The RVM of the benthic short-horned sculpin was 2-fold greater than in the semi-pelagic lumpfish or the very active Atlantic salmon (Table 1). Active fishes generally have a larger RVM than benthic fishes [45]. Nonetheless, an RVM twice that of other cold benthic species (0.06%; [46]) was previously observed in a population of short-horned sculpin endemic to the west coast of Greenland (0.131–0.124%; [47]). Further, these authors showed that the relatively larger heart of sculpin supports an impressive cardiac performance, which is indeed greater than that of several active species like the rainbow trout and Atlantic cod [47]. The RVM of Atlantic salmon and lumpfish was also comparable to that previously reported for these species (0.076% for Atlantic salmon [48] and 0.088% for lumpfish [49]).

### 4.3. Citrate Synthase Activity

The activity of CS in the heart of Mb^−^ lumpfish was similar or greater than that of the other two Mb^+^ species (5.1 µmol g tissue^−1^ min^−1^ vs. 3.8 and 6.5 in the sculpin and salmon, respectively; Table 1). Thus, the loss of Mb in Hb^+^ species was not associated with a reduced aerobic metabolic capacity, and this agrees with the literature [12,17,21]. Interestingly, the expression of Mb was associated with a greater CS activity in Hb^−^ icefishes [50], a trait that does not appear to be shared with Hb^+^ fishes. In our study, CS activity was negatively correlated with these species’ RVM and P:O ratio (Supplementary Figure S3 and Figure 1D), and these data suggest that increased CS activity may be needed in fish with smaller hearts and/or that have a lower efficiency of oxidative phosphorylation. However, measurements of the myocardial oxygen consumption and power output of these hearts need to be performed before the relationship between CS, ventricular size, heart function, and myocardial efficiency can be elucidated.

### 4.4. Relationship between Mb Content and Mitochondrial Function

Differences in mitochondrial respiration and coupling (OXPHOS, LEAK and the resulting RCR) between the species in this study were only observed when stimulating the ETS with CI substrates (Figure 1). This inter-specific variation in CI functionality agrees with CI being an important site of physiological plasticity. For example, inter-specific variation in hypoxia tolerance has been related to the CI capacity of mitochondria in the sculpin brain [51], and this latter parameter was shown to be associated with intra-specific variation in the temperature-dependent mitochondrial function of Atlantic salmon hearts [29,52]. Cardiac mitochondria from lumpfish had OXPHOS-I and RCR-I respiration values that were lower as compared with the high activity Mb^+^ salmon and the sedentary Mb^+^ sculpin, respectively (Figure 1A,C).

S-nitrosation of CI due to the absence of the NO scavenger Mb in lumpfish may contribute to this difference in CI capacity. S-nitrosation of CI in mitochondria from the heart of freshwater turtles (*Trachemys scripta*) decreased ROS production and CI activity [8]. In support of this hypothesis, ROS production (expressed as pmol H_2_O_2_ mg protein^−1^ s^−1^) under OXPHOS-I in lumpfish was slightly (~10%) lower than in the salmon, and significantly lower than in the sculpin (0.185 vs. 0.200 and 0.226 pmol H_2_O_2_ mg protein^−1^ s^−1^, respectively; Figure 2B). In contrast, sculpin mitochondria had the highest ROS production/release rate during LEAK-I and -I+II, and the loss of Mb was not associated with an increased ROS production/release rate under these states (Figure 2). This finding is consistent with the study of O’Brien et al. (2019). These authors showed that variation in Mb expression in five species of notothenioid fishes was not associated with oxidative stress/damage in the ventricle [50]. This may be because inter-specific ROS scavenging by Mb^+^ and Mb^−^ fishes may only be relevant/observed under conditions of limited oxygen. Indeed, high Mb content was previously shown to be beneficial for scavenging ROS and NO under conditions of reduced oxygen supply [53,54].No differences in myocardial oxygen consumption were observed between the two Mb^+^ species and the Mb^−^ lumpfish after addition of the CII substrate succinate [i.e., in OXPHOS-I+II (State 3), LEAK-I+II (State 4) and the resulting RCR-I+II (State 3/State 4)] (Figure 1). Comparable ETS capacity in species that differ in Mb content agrees with a previous study that reported similar activities of key enzymes of energy metabolism in the hearts of the myoglobin-rich sea raven (*Hemitripterus americanus*) and the myoglobin-poor ocean pout (*Macrozoarces americanus*) at high PO_2_ [21]. However, Legate et al. [22] found that while the O_2_ consumption of isolated myocytes was comparable in these two species at high PO_2_s, differences arose at low PO_2_ levels. Therefore, although the P_50_ for mitochondrial respiration is very low (5–20 times less than the half O_2_ saturation point of Mb; [55]), it is possible that the loss of Mb may only be important under low/limiting oxygen (PO_2_) conditions.

We reported P:O ratios of ~3–3.5 with glutamate, malate and pyruvate for the cardiac mitochondria of all three fish species (Figure 1D). These P:O ratios are high as compared to values reported with pyruvate and malate alone (~2.5; [56]), and are likely due to glutamate dehydrogenase generating additional NADH as glutamate is converted to α-ketoglutarate. The amount of substrate-level phosphorylation by glutamate is unknown [56], but in dog mitochondria, glutamate P:O ratios were also over 3 [57]. The P:O ratio for lumpfish was the same as for salmon, which had a nearly identical RVM, and this suggests that oxidative phosphorylation is not influenced by the presence/absence of Mb. These results contrast with that obtained with C2C12 cells derived from mice myoblasts [58], where cells made to overexpress Mb showed an improvement in the efficiency of producing ATP from ADP.

No clear patterns between oxidative capacity, mitochondrial function or oxidative status and Mb content were apparent. This result supports the findings of previous studies which showed that aerobic capacity was comparable amongst low activity species despite great differences in Mb content when measured at high PO_2_ [12,20,21,22].

### 4.5. Relationship between Myoglobin Content and the NOS/NO System

We found a positive relationship between NOS activity and cardiac Mb content when the data for all three species were combined (Figure 5A,B). A similar relationship was previously established between NOS activity and circulating [Hb] in red- and white-blooded Antarctic notothenioid fishes [23]. Hence, there is growing evidence that reduced NOS activity is a common feature of fishes lacking one of the NO scavengers (Hb^−^ and/or Mb^−^). The physiological consequences of a reduction in NOS activity should be further investigated. It is possible that Mb^−^ fish decrease NOS activity to preclude a higher constitutive NO-tone due to the absence of potent NO scavengers. Indeed, a constitutive NO tone has been shown in several red-blooded fish species to affect both basal mitochondrial respiration [14] and myocardial contractility [59]. Furthermore, it remains unclear whether decreasing NOS activity mitigates the accumulation of NOx (nitrite/nitrate) in Hb^−^ Mb^−^ fishes. Plasma nitrite has been shown to reflect NOS activity in mammals, and is recognized as a useful indicator of NOS activity/NO production [60]. However, NOx levels in the plasma of *C. gunnari* (Hb^−^ Mb^+^) were comparable to those in three Hb^+^ notothenioids with higher NOS activity (*L. kempi, N. coriiceps* and *G. gibberifrons*), and even higher plasma NOx was measured in *C. aceratus* (Hb^−^ Mb^−^) despite it having a lower NOS activity [23]. It is possible that NOx in the plasma is influenced by the loss of the circulating NO scavenger Hb, but the influence of Mb concentration on plasma NOx is unlikely. In addition, differences inNOx levels were not investigated here as it can be greatly influenced by a species’ diet, its nitrite susceptibility and concentrations in the environment [61], and this deserves further study.

Importantly, the O_2_ affinity of Mb determines this heme’s function as a NO scavenger and producer, and may result in different NOx levels and effects. This is illustrated by the different amount of nitrite accumulation, and effects, at the same O_2_ tension in the myocardium of trout (low affinity Mb) vs. goldfish (high affinity Mb) [14]. Further, NOx content can also be influenced by the various sources of nitrite in fishes [61], and the up-regulation of other NO/nitrite/nitrate pathways in Mb^−^ and/or Hb^−^ species. In Mb^−^ mice, xanthine oxidoreductase (XOR)-related pathways are up-regulated to compensate for the lack of Mb [2]. In addition, negative-feedback regulation of iNOS (i.e., NOS2) gene expression by nitrite has previously been reported in brown trout [62], and this suggests that fish with higher NO_X_ may have lower NOS activity. To our knowledge, constitutive NO tone and its modulation by NOx level and NOS activity have not been examined in Mb^−^ and Mb^+^ red-blooded fish species. However, differential modulation of basal mitochondrial respiration and cardiac function can be expected. The first evidence of the differential modulation of mitochondrial respiration is the difference in mitochondrial sensitivity to inhibition by NO (NO IC_50_; Figure 4). In this study, a higher concentration of NO was required to reduce mitochondrial respiration by half in the Mb^+^ salmon and sculpin as compared to the Mb^−^ lumpfish (Figure 4 and Figure 5C). This change in mitochondrial sensitivity to NO is in line with the enhanced sensitivity to NO, and activation of downstream pathways that regulate NO, in Mb^−^ mice [2,63,64]. In order to preserve NO and O_2_ homeostasis in the absence of this ‘O_2_ carrier-NO scavenger/producer’, NOS activity and mitochondrial NO sensitivity must be finely tuned. The positive correlation between NO IC_50_ and NOS activity in the ventricle of our fishes support this hypothesis (Figure 5D). Indeed, in the present study, low NO IC_50_ was also associated with low ventricular NOS activity, and these parameters were decreased by 30–40% in the Mb^−^ lumpfish (Figure 4B and Table 2). In addition, our previous study [29] and that of Jørgensen et al. [65] provide compelling evidence that NOS activity and mitochondrial NO IC_50_ in the salmon heart are synchronized/simultaneously adjusted. Specifically, these two studies collectively show that cold-acclimated salmon have low NOS activity and a lower NO IC_50_, whereas warm-acclimated had higher values for these two parameters. The lack of Mb in the lumpfish heart may have required a reduction in NOS activity and an adjustment of the NO IC_50_ to preserve NO homeostasis, and this may be a strategy employed by other Mb^−^ fish to preserve NO homeostasis and the regulation of mitochondrial and cardiac function. NO concentration must be kept under tight control/in a fine balance to exert its physiological function while avoiding toxicity (see [66] for a review ‘on how mammals and fish maintain NO homeostasis, both through NOS enzymes and globins’). Generation of sufficient NO levels to regulate important cardiovascular and mitochondrial functions is crucial, and may explain why an increase in sensitivity to NO (i.e., a lower NO IC_50_) might be of benefit to Mb^−^ fish.

Interestingly, the NO IC_50_ values for the three species appeared to closely match their hypoxia tolerance (critical oxygen tension, P_crit_). For example, the P_crit_ and NO IC_50_ values for the two Mb^+^ species (salmon and sculpin) are 40% of air saturation (air sat.) [67,68,69] and 58 nmol L^−1^ vs. 33% air sat. [44] and 66 nmol L^−1^. Further, the lumpfish is the least hypoxia tolerant of the species studied, with a P_crit_ of 54.4% air sat. [70] and an NO IC_50_ of 44 nmol L^−1^, and we previously measured an NO IC_50_ value of 126 nmol L^−1^ in sablefish (*Anoplopoma fimbria*) [28], a very hypoxia tolerant species with a P_crit_ of 16% air sat. [71]. Hence, mitochondrial sensitivity to NO (i.e., its IC_50_) may also be a good marker/indicator of a species hypoxia tolerance. This hypothesis is not surprising as both NO and Mb (and their interactions) play a critical role in fish hypoxia tolerance [72,73].

No significant relationships could be established between NOS activity measured in the mitochondrial suspensions and mitochondrial NO IC_50_ or ventricular NOS activity (Figure 5E,F). The existence of mitochondrial NOS (mtNOS), identified as a nNOS by most studies, and its functional role in regulating mitochondrial function is still being debated [74,75,76,77]. Mitochondrial NOS activity in our Hb^+^ Mb^+^ fish (0.15–0.10 nmol mg protein^−1^ min^−1^) is slightly lower than that measured in porcine heart mitochondria (0.22 nmol mg protein^−1^ min^−1^; [78]). We, however, cannot rule out that contamination of the mitochondrial preparation with cytosolic NOS resulted in ‘apparent’ mtNOS activity. Whether mitochondrial NOS is a significant source of NO in the fish heart, and modulates oxidative phosphorylation, should be confirmed by evaluating the effects of substrates and inhibitors of NOS on mitochondrial respiration at low (physiologically relevant) O_2_ levels [as NO is outcompeted for binding to CIV at high O_2_/in air-saturated solutions].

Studies on red-blooded fishes with differing cardiac Mb levels may help to improve our understanding of the role of Mb in NO homeostasis and signalling, and how they mediate mitochondrial and cardiac function. Overall, our data support the idea that compensatory mechanisms are at play in Hb^+^ fishes that preserve NO and O_2_ homeostasis, as reported in Mb^−^ mice [2] and in Hb^−^ icefishes [24]. In red-blooded fishes, the loss of Mb was associated with lower NOS activity but an increased sensitivity to NO (a lower IC_50_; at least at the mitochondrial level). These data support the concept that NO is a ‘master controller’ of physiological traits in species lacking globins [17], and that globins play a critical role in NO homeostasis [66].

## 5. Conclusions

This study explored whether the presence/absence of Mb correlates with oxidative capacity and aspects of the NOS/NO system in the myocardium and mitochondria of red-blooded fish species. Overall, we report a reduction in NOS activity and an increase in the sensitivity to NO (at least at the mitochondrial level) in the Mb^−^ lumpfish as compared to the Mb^+^ salmon and sculpin. These adjustments in mitochondrial sensitivity to NO and cardiac NOS activity likely compensate for the loss of Mb (an important NO scavenger and producer in cardiac tissue), and ensure that mitochondrial function is finely tuned under various conditions. This comparative study using Hb^+^ species with differing Mb content adds greatly to our knowledge of the dynamic and complex relationships between globins and NO, and adds further insights into how the heart deals with limitations in oxygen supply.

## Figures and Tables

**Table 1:** Ventricular characteristics of the experimental species.

	*S. salar*	*M. scorpius*	*C. lumpus*
Relative ventricular mass (%)	0.075 ± 0.004 ^a^	0.149 ± 0.010 ^b^	0.079 ± 0.004 ^a^
Myoglobin content (nmol g tissue^−1^)	26.49 ± 4.6 ^a^	20.05 ± 2.1 ^a^	ND ^b^
Citrate synthase activity (µmol g tissue^−1^ min^−1^)	6.48 ± 0.34 ^a^	3.81 ± 0.23 ^b^	5.08 ± 0.23 ^c^

Values are means ± s.e.m., *n* = 7 per group; except for Mb content where *n* = 8–11 per group (Mb content was measured in additional fishes due to the magnitude of intra-individual variability). Values without a letter in common are significantly (*p* < 0.05) different; one-way ANOVAs followed by Tukey’s HSD multiple comparison tests. ND, not detectable.

**Table 2:** Nitric oxide synthase (NOS) activity in the ventricle and ventricular mitochondria of the three experimental species.

	*S. salar*	*M. scorpius*	*C. lumpus*
Ventricular NOS activity (nmol g tissue^−1^ min^−1^)	3.30 ± 0.24 ^a^	3.90 ± 0.27 ^a^	2.17 ± 0.15 ^b^
Mitochondrial NOS activity (nmol mg protein^−1^ min^−1^)	0.15 ± 0.010 ^a^	0.10 ± 0.017 ^b^	0.04 ± 0.004 ^c^

Values are means ± s.e.m., *n* = 7 per group; except for mitochondrial NOS activity where some samples were pooled (2 to 4 individuals) prior to measurement, and activity was measured in additional fishes; *n* = 6–8. Values without a letter in common are significantly (*p* < 0.05) different; one-way ANOVAs followed by Tukey’s HSD multiple comparison tests.

## Data Availability

The data presented in this study are available on request from the corresponding author.

## Supplementary Materials

The following are available online at https://www.mdpi.com/article/10.3390/antiox10071072/s1. Figure S1: Representative recording of Experiment 1. Figure S2: Representative recording of Experiment 2. Figure S3: Relationships between ventricular citrate synthase (CS) activity, relative ventricular mass (RVM) and mitochondrial efficiency (P:O ratio) of Atlantic salmon, short-horned sculpin and lumpfish.
