# Supplementary material for: The Relationship between Myoglobin, Aerobic Capacity, Nitric Oxide Synthase Activity and Mitochondrial Function in Fish Hearts
Source: Antioxidants (Basel). 2021 Jul 3;10(7):1072. doi: 10.3390/antiox10071072 (PMC8301165; doi:10.3390/antiox10071072)
Supplement: Supplementary file 1 [file antioxidants-10-01072-s001.zip › antioxidants-1264514-supplementary.pdf]

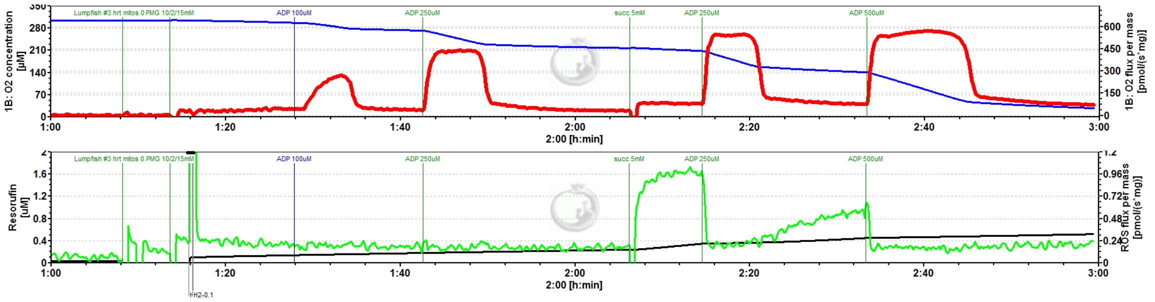

Supplementary Figure S1

Supplementary Figure S1. Representative recording of Experiment 1. The figure shows the simultaneous measurement of O_2 consumption (flux) and ROS release rate (flux) during OXPHOS (State 3) and leak (State 4) respiration in the presence of substrates of complex-I alone (pyruvate, malate and glutamate) and complexes-I+II (+ succinate). O_2 concentration (in µM) is represented by the blue line, O_2 flux [i.e., mitochondrial respiration rate (in pmol O_2 $(s*mg)^{-1}$)] by the red line, H_2O_2 concentration (in µM) by the black line and H_2O_2 flux [i.e., mitochondrial ROS release rate (in pmol H_2O_2 $(s*mg)^{-1}$)] by the green line. Events and concentrations of chemical used are labeled on the recording. Figure shows data from cardiac mitochondria (0.25 mg mL^{-1} chamber) of lumpfish incubated at 12°C.

Supplementary Figure S2

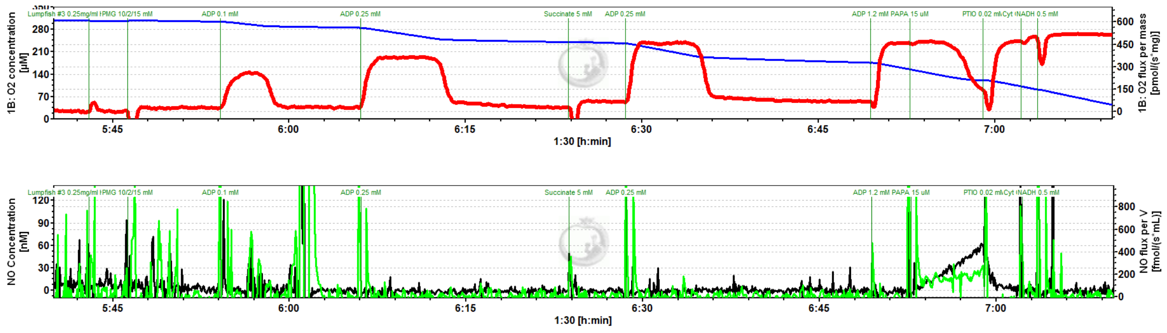

Supplementary Figure S2. Representative recording of Experiment 2. The figure shows the measurement of mitochondrial respiration during OXPHOS (State 3) in the presence of substrates of complexes-I+II (pyruvate, malate, glutamate and succinate, PMGS) and excess ADP; following the initiation of NO production (using the NO donor PAPANONOate); after PTIO was added to the chamber to scavenge NO and re-establish OXPHOS respiration; and finally after cytochrome c and NADH were added to examine mitochondrial integrity. This protocol was used to determine the sensitivity of mitochondrial respiration to NO (i.e., its IC_{50}). O_2 concentration (in µM) is represented by the blue line, O_2 flux [i.e., mitochondrial respiration rate (in pmol O2 $(s*mg)^{-1}$) by the red line, NO concentration (in µM) by the black line and NO flux (in fmol NO $(s*mg)^{-1}$) by the green line. Events and concentrations of chemical used are labeled on the recording. Figure shows data from cardiac mitochondria (0.25 mg mL^{-1} chamber) of lumpfish incubated at 12°C.

Supplementary Figure 3

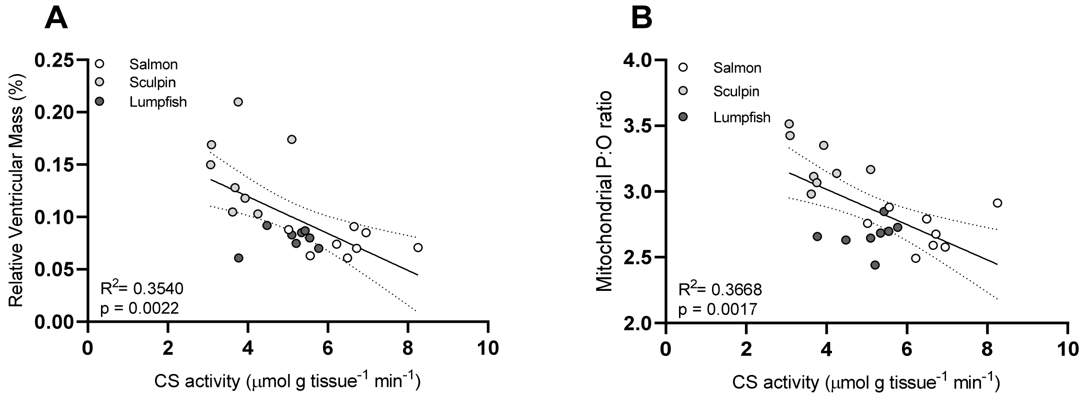

Supplementary Figure S3. Relationships between ventricular citrate synthase (CS) activity, and relative ventricular mass (RVM) and mitochondrial efficiency (P:O ratio), of Atlantic salmon, short-horned sculpin and lumpfish. The solid line represents the overall linear pairwise regression and the dotted lines represent the 95% confidence limits of the fitted line. Symbols represent individual paired values. Values are means ± s.e.m. (N=8 per group). A $P<0.05$ was considered significant.
